# Shelf-inflicted head injuries

**DOI:** 10.1007/s10140-025-02430-6

**Published:** 2026-01-15

**Authors:** Nitin Menon, Fatima Mohamed, Rachael Hutchinson, Rob A. Dineen

**Affiliations:** 1 Radiology Department, Queen’s Medical Centre, Nottingham University Hospitals NHS Trust, Nottingham, United Kingdom; 2 Radiological Sciences, Mental Health and Clinical Neuroscience, School of Medicine, University of Nottingham, Nottingham, UK; 3 Sir Peter Mansfield Imaging Centre, University of Nottingham, Nottingham, UK; 4 NIHR Nottingham Biomedical Research Centre, Nottingham, UK

**Keywords:** Head injury, Minor head trauma, Computed tomography, Diagnostic yield, Emergency radiology, NICE guidelines

## Abstract

**Purpose:**

To evaluate the diagnostic yield of cranial computed tomography (CT) imaging in patients who sustained minor head trauma after directly hitting their head on a shelf or cupboard, without an associated fall. The study aimed to assess whether such low-impact mechanisms warrant cranial imaging.

**Methods:**

This was a retrospective observational study conducted at the emergency department of a major trauma centre in the United Kingdom. Data were extracted from the institutional Radiology Information System and electronic health records, covering the period from June 2006 to April 2025. An initial cohort of 320 patients referred for CT head imaging was identified using a keyword-based search of radiology request histories. Of these, 109 patients were included based on a clearly documented mechanism of injury involving impact with a cupboard or shelf, without an associated fall. Exclusion criteria included alternative mechanisms such as falls, falling objects, assault, or irrelevant mentions. The primary outcome was the presence of acute intracranial pathology on CT head. Secondary data collected included age, sex, injury mechanism, clinical symptoms, adherence to National Institute for Health and Care Excellence (NICE) head injury imaging criteria, and any scalp or skull findings.

**Results:**

Of the 109 included patients, median age was 48 (31–66), females 76 (70%). Eight patients (7%) had scalp findings, including swelling or laceration. Despite the low-force mechanism, 79 patients (72%) met at least one NICE guideline criterion for imaging, most commonly due to vomiting or anticoagulant use. None demonstrated acute intracranial pathology or skull fracture on the CT head scan. The absence of positive findings suggests that careful vetting of NICE criteria in the context of biomechanically implausible mechanisms may reduce unnecessary imaging.

**Conclusion:**

Walking into or standing up and hitting a shelf or cupboard is an insufficient force to generate clinically significant traumatic brain injury. Direct head impact from a fixed object, without an associated fall, appears to be a biomechanically low-risk mechanism. This study supports a more scrutinised and context-aware application of the NICE head injury criteria in such scenarios, to help rationalise imaging demand, optimise resource use, and reduce unnecessary exposure to ionising radiation.

**Graphical Abstract:**

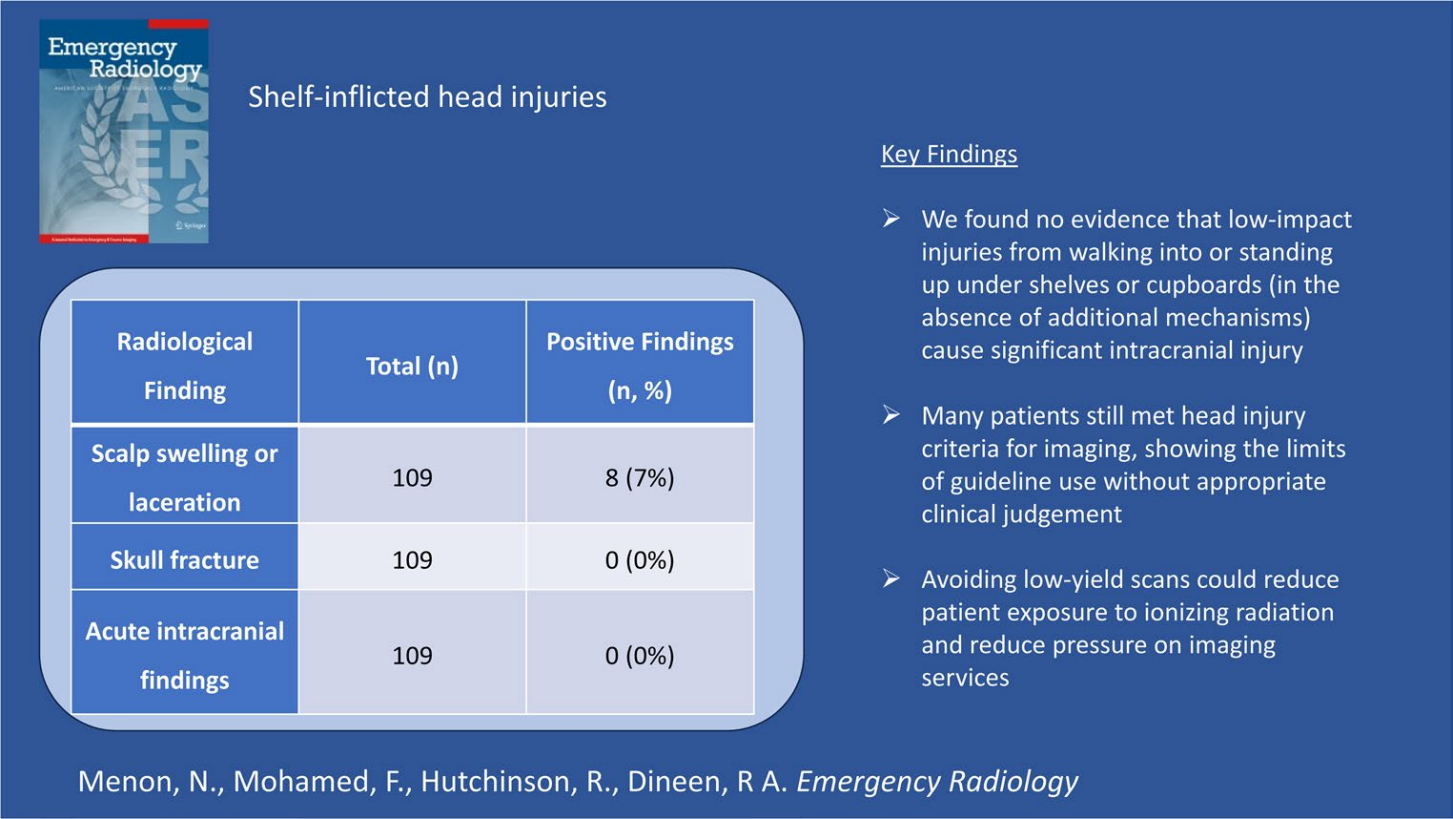

## Introduction

Head trauma represents a substantial proportion of emergency department attendances, with approximately 90% of cases classified as minor head injuries [1, 2]. Despite the low probability of significant intracranial pathology in these patients, CT imaging is frequently overused. Studies have shown that a considerable number of CT head examinations are performed outside of established clinical guidelines, particularly in younger patients and in cases involving low-risk mechanisms of injury [3]. This trend highlights the importance of rigorous guideline adherence to ensure appropriate resource use and minimise unnecessary exposure to ionising radiation. National guidance, including the NICE Head Injury guideline NG232, advises cranial CT imaging only for patients meeting clearly defined risk criteria, such as loss of consciousness, post-traumatic amnesia, vomiting, seizure activity, or anticoagulant use [2]. For patients who do not meet these thresholds, observation is recommended as a safe alternative.

In clinical practice, however, the threshold for requesting CT imaging is often lower than suggested by guidelines [3]. Anecdotally, and particularly in acute or out-of-hours settings, CT head scans are frequently performed in patients who do not fulfil NICE criteria. This tendency is influenced by risk aversion, time pressures, diagnostic uncertainty, and medico-legal concerns. Cranial CT has become a disproportionately large component of the acute neuroradiology workload. CT head examinations now constitute a significant proportion of out-of-hours imaging activity, with a 15-year retrospective study showing a tenfold increase in overnight CT volume [4]. CT head was the most frequently performed examination type, highlighting its dominant role in after-hours radiology demand [4]. This practice, while driven by caution, has significant implications, including increased demand on radiology resources, avoidable healthcare costs, and patient exposure to ionising radiation [5, 6]. 

**Fig. 1:**
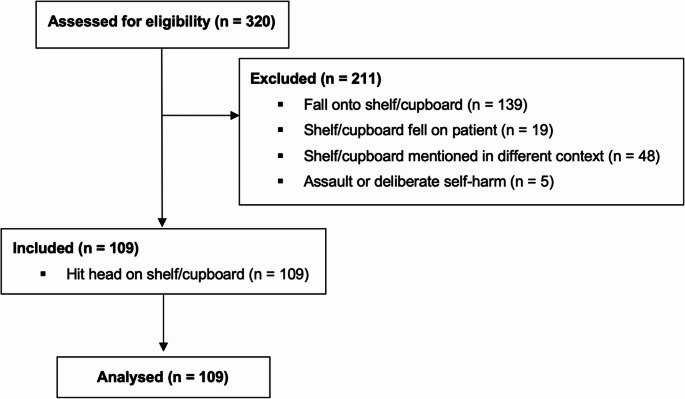
Flow diagram of case selection

A specific and under-evaluated example of low-impact trauma is the scenario in which patients hit their head on a cupboard or shelf, either by walking into it or standing up beneath it, without any associated fall (Fig. 1). These mechanisms are mechanically constrained; the force imparted is minimal, with negligible acceleration or deceleration. Compared with high-risk mechanisms such as falls from height, motor vehicle collisions, or assaults, the energy transfer is limited. Consequently, it is biomechanically implausible that such isolated impacts would result in skull fracture or intracranial haemorrhage in the absence of additional risk factors. Inertial loading, that is, rapid linear or rotational acceleration of the head, is the primary driver of intracranial injury, rather than static contact alone [7].

Despite this, CT imaging is sometimes requested for these patients. This retrospective evaluation aims to quantify the diagnostic yield of CT head imaging in this specific group, using data from a UK Major Trauma Centre. Our objective is to evaluate whether this mechanism justifies routine cranial CT and to support more targeted, evidence-based imaging practice.

## Methods

This retrospective observational study was conducted at a UK Major Trauma Centre to evaluate the diagnostic yield of cranial CT imaging in patients presenting with head trauma resulting specifically from contact with a shelf or cupboard. The study aimed to determine whether this low-impact mechanism, in the absence of a secondary fall, is sufficient to result in clinically significant intracranial injury.

Cases were identified from the Radiology Information System (RIS) of Nottingham University Hospitals NHS Trust. All cranial CT requests performed between June 2006 and April 2025 were searched using a text-based keyword strategy targeting the terms “cupboard” and “shelf” within the clinical history field of the scan request. This search yielded 320 cases. Each case was reviewed in full to determine mechanism of injury and to assess eligibility.

Inclusion was restricted to cases where the history explicitly described the patient having hit their head on a shelf or cupboard, either by standing up into it or walking into it, without subsequently falling to the ground. Cases were excluded if the mechanism involved a fall onto a cupboard or shelf, if the object fell onto the patient, if the cupboard or shelf was mentioned only in a non-traumatic context (e.g. location of injury), or if the incident was the result of assault or deliberate self-harm. After applying these criteria, 109 cases were retained for final analysis.

Clinical data were collected through structured review of the Digital Health Record (DHR) and radiology reports. Variables included patient age and sex, mechanism subtype, symptoms at presentation (including headache, dizziness, nausea or vomiting), use of anticoagulation therapy, documented loss of consciousness, and fulfilment of NICE 2023 guideline criteria for CT head. Clinical examination findings were also recorded, including Glasgow Coma Scale (GCS) score, presence of scalp swelling, lacerations, palpable skull defects, and any documented neurological deficits.

Radiological findings were extracted from formal CT reports. These were categorised as scalp soft tissue injury (swelling or laceration), skull fracture, or acute intracranial pathology (such as subdural or epidural haematoma, subarachnoid haemorrhage, or contusions). The primary outcome measure was the presence of any acute intracranial findings on CT scan.

Descriptive statistics were used to summarise the cohort. For continuous variables, median and interquartile range (IQR) were reported; for categorical variables, frequencies and percentages were calculated. All analyses were conducted using Microsoft Excel. An analysis plan was pre-specified made openly available prior to data analysis commencing [8]. The analysis, conducted as a service evaluation using existing clinical data, did not require Research Ethics Committee review as per UK Health Research Authority guidance [9]. Patients and the public were not involved in the design, conduct, reporting, or dissemination of this research, as the study was a retrospective service evaluation using existing clinical data.

## Results

A total of 320 cranial CT referrals were identified using keyword-based searches of the Radiology Information System (RIS). Following manual review, 211 cases were excluded based on mechanism of injury or clinical context. These included 139 cases involving a fall onto a shelf or cupboard, 19 where a cupboard or shelf fell onto the patient, 48 with irrelevant mentions of a shelf or cupboard, and 5 related to assault or deliberate self-harm. The final study cohort comprised 109 patients whose injuries were due to direct impact against a shelf or cupboard, either by standing up into or walking into it, without a secondary fall. The selection process is summarised in Fig. 1.

Demographic data for the included patients are summarised in Table 1. The median age was 48 years (31 to 73), with a predominance of female patients (76/109, 70%).

**Table 1:** Demographic characteristics

Characteristic	Value
Total patients	109
Age (median, IQR)	48 years (31–73)
Female sex	76 (70%)

Clinical presentation data were variably reported (Table 2). Among the 93 cases with available information on headache, 63 (68%) described headache beyond localised pain at the impact site. Nausea and/or vomiting were documented in 55 of 97 cases (57%), while Dizziness was noted in 34 of 90 (38%). Loss of consciousness was recorded in 18 of 91 (20%) and anticoagulation use in 22 of 83 cases (27%). Based on available clinical information, 79 of the 109 patients (72%) fulfilled at least one of the 2023 NICE criteria for cranial CT imaging. Examination findings were infrequently documented. Scalp swelling was recorded in 14 of 81 patients (17%), and lacerations in a similar proportion. Palpable skull defects and GCS abnormalities were rare, reported in 3 (4%) and 7 (9%) of those with documentation, respectively.

**Table 2:** Clinical features at presentation

Clinical Characteristic	Total Reported (*n*)	Present (*n*, %)
Headache	93	63 (68%)
Dizziness	90	34 (38%)
Nausea and/or vomiting	97	55 (57%)
Loss of consciousness	91	18 (20%)
Anticoagulation	83	22 (27%)
Fulfilled NICE criteria	109	79 (72%)
Scalp swelling	81	14 (17%)
Palpable skull defect	82	3 (4%)
Laceration	81	14 (17%)
GCS deficit	74	7 (9%)

Radiological findings are shown in Table 3. Scalp soft tissue injuries (swelling or laceration) were present on imaging in 8 patients (7%). There were no cases of skull fracture or acute intracranial pathology in the final study cohort (0%, 0/109).

**Table 3:** CT head outcomes

Radiological Finding	Total (*n*)	Positive Findings (*n*, %)
Scalp swelling or laceration	109	8 (7%)
Skull fracture	109	0 (0%)
Acute intracranial findings	109	0 (0%)

## Discussion

In this cohort of 109 patients who sustained head trauma from walking into or standing up under a cupboard or shelf without an associated fall, there were no cases of acute intracranial pathology identified on CT. This suggests that such mechanisms, while often prompting concern for patients and the clinical team, are very unlikely to result in clinically significant injury.

The majority of patients in our cohort (72%) met one or more criteria outlined in the 2023 NICE guideline on head injury (NG232), which recommends CT imaging based on clinical features such as vomiting, loss of consciousness, or anticoagulant use [2]. While these criteria provide a valuable risk stratification framework, their application may be overly inclusive in certain contexts. Specifically, our findings highlight the need to consider the plausibility of the trauma mechanism itself. Impacts to the head without associated acceleration or deceleration forces, as seen when a person stands into or walks into a fixed object, impart low kinetic energy and are unlikely to produce sufficient brain strain to cause haemorrhage or fracture. This is supported by biomechanical studies demonstrating that inertial loading, rather than static contact, is typically responsible for serious intracranial injury [10]. 

Other studies have similarly suggested that adherence to guidelines, while generally safe, may lead to imaging in scenarios with very low diagnostic yield when applied uncritically. Imaging patterns and outcomes vary significantly depending on trauma mechanism, with high-velocity or fall-related injuries carrying substantially more risk than static blunt impacts [11]. 

In clinical practice, CT imaging for minor head injury is often initiated pre-emptively, even in cases where guideline criteria are not strictly met. Empirical studies have identified key drivers of imaging overuse in the emergency department, including physician fear of missing diagnoses, concern over litigation (defensive medicine), perceived patient expectations, and organisational pressures such as time demands and workflow efficiency [3, 12]. Once a decision to scan has been made, NICE criteria may be applied retrospectively or used loosely to justify the request. These patterns highlight the importance of sharper clinical scrutiny. Both clinicians requesting scans and radiologists vetting them should assess not only whether formal criteria are met, but also whether the described mechanism of injury plausibly supports a risk of intracranial injury.

The increasing demand for acute CT imaging places further emphasis on this issue. CT head is widely recognised as one of the most frequently requested imaging studies in acute settings, particularly during out-of-hours periods, and constitutes a substantial portion of the emergency and inpatient radiology workload [4]. With constrained workforce resources and growing patient volumes, ensuring imaging is reserved for cases with meaningful diagnostic likelihood is essential. Overuse of CT not only strains capacity but also exposes patients to unnecessary ionising radiation. While the absolute risk from a single scan is low, repeated exposure across populations can have public health implications, particularly in younger patients [5]. Rising imaging volumes, particularly in acute and out-of-hours settings, have been consistently associated with radiologist stress, reduced job satisfaction, and burnout. Workforce analyses from the UK and international studies demonstrate that increasing workload intensity, overnight reporting burden, and sustained service pressure contribute to fatigue and reduced wellbeing among radiologists [13–15]. Reducing low-yield or unnecessary imaging, especially in clearly low-risk scenarios, represents one practical way to mitigate this pressure and redirect limited radiology capacity towards examinations with greater diagnostic and clinical value.

This study has limitations. It was retrospective and conducted at a single centre, with a relatively small sample size of 109 patients identified over a 10-year period. The low number of cases limits statistical power and restricts the generalisability of findings. Although this mechanism represents only a small fraction of the total cranial CT workload, its evaluation remains clinically relevant. To our knowledge, this is the first study to specifically investigate the diagnostic yield of CT head imaging in cases involving direct head impact from walking into or standing up beneath a cupboard or shelf. While clinical records were reviewed in detail, the accuracy and completeness of documented histories cannot be fully assured. Broader search terms such as “door” or “table” may have increased case yield but at the cost of specificity, requiring extensive filtering. Future studies could examine a wider range of low-impact mechanisms across multiple centres. Additionally, imaging findings were extracted from radiology reports rather than direct image review, which may limit sensitivity for subtle findings. However, as all reports were verified by consultant neuroradiologists, the data are considered sufficiently robust for the purposes of this study. Minor findings such as subtle scalp swelling may still be underreported due to variability in routine reporting. Nonetheless, the consistent mechanism and absence of acute intracranial pathology support the conclusion that such injuries carry extremely low risk.

These findings support a more nuanced application of national guidelines, advocating for mechanism-based reasoning alongside symptom checklists. In the context of limited radiology resources, careful scrutiny of both clinical features and biomechanical plausibility may help reduce unnecessary imaging, streamline emergency care, and safeguard patients from avoidable radiation.

## Data Availability

Tabulated, fully anonymised data that support the findings of this study are available from the authors on reasonable request.
